# Achieving immunization milestones: Insights from Oman’s national coverage survey

**DOI:** 10.1371/journal.pone.0327788

**Published:** 2025-07-10

**Authors:** Bader Al-Rawahi, Prakash K. P., Noura Al-Farsi, Mariyam Al-Shaibi, Athari Al-Jahwari, Bader Al-Abri, Seif Al-Abri

**Affiliations:** 1 Department of Communicable diseases, Ministry of Health, Centre for disease control and prevention, Muscat, Oman; 2 Department of Surveillance, Ministry of Health, Centre for disease control and prevention, Muscat, Oman; 3 Ministry of Health, Directorate. Centre for disease control and prevention, Muscat, Oman; Central Laboratory for Evaluation of Veterinary Biologics, Agricultural Research Center, EGYPT

## Abstract

The studies primary objective was to determine the actual immunization coverage by validating routine immunization data and conducting a community-level survey at the sub-national level for different age groups in Oman. A cross-sectional community survey to assess vaccination coverage in Oman was done using the WHO cluster sampling method on children under five eligible for various vaccines, including BCG, HBV, OPV, PCV, Penta, Hexa, MMR, Varicella, and Hepatitis A. The survey was conducted between 20^th^ February 2023 and 19^th^ March 2023. Face-to-face interviews using a pretested electronic questionnaire gathered the information. Descriptive statistics and chi-square tests for categorical data analysis were performed. Vaccination outcomes were classified as fully vaccinated, partially vaccinated, or unvaccinated, with 95% confidence intervals reported. The crude national vaccination coverage was nearly 100% in Oman. The fully vaccinated coverage ranged from 87% to 99%. Only one child (0.01%) was unvaccinated, and 1.3% of children were partially vaccinated. Children in Muscat and Dhofar had lower odds of being fully vaccinated compared to other governorates and Omani children were significantly more likely to be fully vaccinated compared to non-Omani children. There was a difference in the crude coverage and valid coverage indicating there is a delay in vaccination (>30 days from the scheduled date) as the age progresses. The national immunization coverage fully complied with the administrative coverage. The high vaccination coverage indicates universal acceptance of vaccination program in Oman. Delayed vaccination due to acute illness around the due date of vaccination was a significant observation that needs attention.

## Introduction

Globally, immunization is recognized as one of the most effective public health interventions, saving millions of lives from vaccine-preventable diseases (VPDs) each year. The World Health Organization (WHO) estimates that vaccination currently prevents nearly 3.5–5 million deaths annually, attributed to diseases such as diphtheria, tetanus, pertussis, influenza, and measles [1,2].

Oman’s immunization program gained major traction with the formal launch of the Expanded Program on Immunization (EPI) in 1981. Significant progress was made in 1987 with the implementation of standardized record systems like child health cards and registers, leading to immunization coverage exceeding 95% by the early 1990s. These efforts significantly reduced or eliminated many VPDs. Oman has maintained freedom from diseases such as poliomyelitis, neonatal tetanus, diphtheria, and rabies for over two decades and eliminated measles and rubella in 2019 [3]. Over the past four decades, the immunization schedule expanded to cover 13 antigens currently. In 2016, Oman achieved a 99% score in global Effective Vaccine Management (EVM) assessments, recognizing its strong and quality public health infrastructure [4,5].

To achieve the goal of eliminating measles and rubella, vaccine coverage for two doses must be > 95%. In 2019, Oman reached this milestone, as it was officially certified by the WHO Regional Office for the Eastern Mediterranean. This certification underscores Oman’s high vaccine coverage and the quality of its vaccination programs, reflecting the effectiveness of its public health strategies and commitment to maintaining high immunization standards [3,6,7].

Despite progress in immunization efforts, gaps between reported and actual vaccination coverage highlight the need for validation through population-based surveys. This has become increasingly crucial post-COVID-19, given concerns about disruptions in vaccination rates among children under five. These surveys are essential for accurately assessing vaccination coverage in the community, identifying barriers to immunization, and guiding public health strategies to sustain high vaccination rates across different demographic groups [8].

According to the National Immunization Program of Oman, the following vaccines are given free of charge to every child currently. At birth: Bacillus Calmette-Guérin (BCG) and Hepatitis B Vaccine (HBV), at 2 months: Hexavalent [Diphtheria Pertussis Tetanus (DTP), Hepatitis B and Haemophilus Influenza Type B (Hib), Inactivated Polio vaccine (IPV)] Vaccine (Hexa)1 and Pneumococcal Vaccine (PCV)1, at 4 months: Hexa2 and PCV2, at 6 months: Pentavalent [DTP, Hepatitis B and Haemophilus Influenza Type B] vaccine (Penta) and Oral Polio Vaccine (OPV), at 12 months: Measles Mumps Rubella (MMR)1 and Varicella/Chickenpox, at 13 months: Penta and PCV booster, at 18 months: MMR2, Hepatitis A 1^st^ dose and OPV Booster, at 24 months: Hepatitis A 2^nd^ dose [4].

The Sultanate of Oman is located on the southeastern coast of the Arabian Peninsula in West Asia, overlooking the mouth of the Persian Gulf. It shares land borders with Saudi Arabia, the United Arab Emirates, and Yemen. Muscat, the capital and largest city, is the political and economic hub. As of 2024, Oman has a population of approximately 5.28 million. The country is divided into 11 governorates, further subdivided into 61 wilayats (districts) [9].

This paper presents findings from Oman’s national immunization coverage survey, validating routine immunization data, identifying coverage gaps, and informing strategies to sustain high vaccination rates and address barriers to immunization.

## Materials and methods

This cross-sectional study assessed vaccination coverage in Oman, covering all 11 provinces and 61 districts, and targeting 415,791 children under five. A WHO cluster sampling method was used to estimate coverage of ~95% with a precision of ±5% and a 95% confidence interval (CI) (α = 0.01). The required sample size was calculated at 16,470 children, based on an intra-cluster correlation coefficient (ICC) of 0.167 (design effect = 1.67) and an inflation factor of 15%, resulting in 3,294 clusters with five children each [10,11]. Households were proportionally distributed according to district populations and selected systematically, focusing on children under five eligible for various vaccines, including BCG, HBV, OPV, PCV, Penta, Hexa, MMR, Varicella, and Hepatitis A. The survey was conducted between 20^th^ February 2023 and 19^th^ March 2023. Face-to-face interviews using a pretested electronic questionnaire gathered demographic and immunization information and reasons for vaccination failures in the “epicollect5” mobile application (https://five.epicollect.net/project/nic-survey). Vaccination dates were recorded using immunization cards (pink card) available at the house and uploaded in the epicollect5 application. In the absence of the pink card at home, the vaccination dates were recorded from the local health institution records (Child health registry).

This survey utilized a population-based cluster sampling method to ensure comprehensive data collection across various districts (wilayats), including remote and hard-to-reach areas. Clusters were randomly selected using area or block numbers, with a predetermined list prepared before field visits. Data collection began with a randomly chosen first house in each cluster, followed by a systematic selection of every alternate house until at least five interviews per cluster were conducted. Each household interview included one to two children under five, with preference for children under three. If no eligible child was present, the survey moved to the next house. In urban areas with multi-family apartment buildings, a floor was selected at random, and apartments were chosen using a systematic approach. Each complex was treated as a single household for data collection. To ensure data quality, a regional EPI supervisor monitored the process, while a national data manager reviewed data consistency and accuracy through an online system.

The study’s data was refined and analyzed using Microsoft Excel and SPSS Version 21.0, employing descriptive statistics and chi-square tests for categorical data analysis by age, gender, nationality, and governorate and district. Vaccination outcomes were classified as fully vaccinated, partially vaccinated, or unvaccinated, with 95% confidence intervals reported. A fully immunized child is one who has received all the required antigen doses according to the age-specific schedule outlined in Oman’s current immunization program. District-wise coverage was detailed, and significant factors from bivariate analysis were further examined using multivariate logistic regression. Statistical significance was set at p < 0.05, and WHO (2018) guidelines were used for key operational definitions such as crude immunization coverage, valid coverage, timeliness, full immunization coverage and dropout rate [10].

### Ethical approval

The study utilized anonymized disease surveillance and immunization record data, derived from secondary data extracted from the national immunization coverage survey. Therefore, ethical approval was not required, in accordance with the Ministry of Health Oman’s national guidelines, which mandate Health Studies and Research Approval Committee (HSRAC), Oman approval only for studies involving patient interventions. This study adheres to the Helsinki Declaration and complies with the Internal Review Committee guidelines.

## Results

The national immunization coverage survey in Oman included 17,930 children, with 239 excluded for being over the age limit (>5 years), 36 excluded due to inaccessibility (e.g., non-responder), and 154 for data validity issues such as inconsistent records or invalid card uploads. This left 17,501 participants for final analysis.

The survey successfully covered all 3,294 planned clusters and reached 3,352 clusters overall, achieving 101.7% coverage. The survey visited 14,133 households, exceeding the expected 5,289, averaging 4 households per cluster and 1 child per household. Each cluster included an average of 5 children. The distribution of clusters, households, and target children was consistent across all provinces. The survey achieved slightly higher numbers than initially planned as shown in Table 1.

**Table 1:** Basic characteristics of the sample in the national immunization coverage survey according to governorate.

Governorate/ Province	Clusters/ Blocks	Households	Children	Number of Households Per Cluster	Number of Children Per Cluster	Number of Children Per Household
**Buraimi**	74	303	399	4.1	5.4	1.3
**Dakhiliyah**	482	2034	2568	4.2	5.3	1.3
**Dhahira**	186	716	958	3.8	5.2	1.3
**Dhofar**	270	1108	1384	4.1	5.1	1.2
**Musandam**	33	141	185	4.3	5.6	1.3
**Muscat**	744	3232	3709	4.3	5.0	1.1
**North Batinah**	637	2618	3476	4.1	5.5	1.3
**North Sharqiyah**	234	982	1253	4.2	5.3	1.3
**South Batinah**	375	1528	1869	4.1	5.0	1.2
**South Sharqiyah**	285	1307	1499	4.6	5.3	1.1
**Wusta**	32	164	201	5.1	6.3	1.2
**Total**	3,352	14,133	17,501	4.2	5.2	1.2

The survey showed that most respondents were mothers (72.4%), with the majority of vaccinations given at government facilities (98.9%). The leading reason for not receiving vaccinations was medical, such as acute illness around the due date (50.7%). Almost all children (99.4%) had their immunization cards available during the survey. Most children surveyed (66.4%) were aged 24–59 months. The gender distribution was nearly balanced, with 51.2% males and 48.8% females. Omani children represented 97.3% of the sample, while non-Omani children made up 2.7%. The mean and median age was 2.7 ± 1.3 years and 2.7 years respectively, with an age range from 4 months to 5 years.

The overall crude national vaccination coverage was nearly 100% at birth for BCG and HBV vaccines (99.9%, CI: 99.99–1.00). Coverage remained high at 6 months for Penta and OPV vaccines (99.9%, CI: 99.8–99.9) and at 12 months for MMR1 and Varicella (99.5%, CI: 99.3–99.5). Coverage for vaccinations at 18 months (MMR2, DTP & OPV Booster) and 24 months showed a slight decline, with 98.9% (CI: 98.7–99.1) and 94.4% (CI: 93.9–94.8) respectively. The lower coverage of the Hepatitis A vaccine at 24 months may be attributed to its recent introduction in January 2020.

The PCV 1 and 2 dose coverage was 99.9% (17479/17,496). PCV booster dose coverage is 98.9% (13,444/13,586). The PCV coverage at 2 and 4 months was similar to Penta vaccination received at 6 months of age. Similarly, PCV coverage at 18 months was similar to MMR2 vaccination received at 18 months of age. This is due to the effective implementation of MoH policy of verifying earlier doses before the child is given the scheduled vaccine.

For the 2021 birth cohort, coverage at 18 and 24 months was 97.0 and 84.4% respectively, which was marginally lower than in other birth cohorts (2018–2020). Similarly, the 2022 birth cohort saw 93.5% coverage at 12 months when compared to other years. Additionally, around 97% of children born in 2022 were partially vaccinated, a slight decrease from the approximately 99% coverage seen in other cohort years.

Table 2 highlighted vaccination coverage across different demographic categories such as age, gender, nationality, and governorate. The fully vaccinated coverage ranged from 96.2% to 99.6%. Overall 98.7% of children under five had received all the required vaccinations for their age, indicating a high full vaccination coverage. Only one child (0.01%) was unvaccinated, and 1.3% of children were partially vaccinated, meaning they had missed at least one dose of vaccination for their age.

**Table 2:** Fully and partially vaccinated children coverage according to age, gender, nationality and governorate.

Variable	Total Subjects	Fully Vaccinated^a^		Partially Vaccinated		Unvaccinated	
		No.	%	No.	%	No.	%
Age range* **X**^**2**^**: 225.3, df = 3, p < 0.001**							
**< 12 months**	1453	1447	99.6	5	0.3	1	0.1
**12-18 Months**	2252	2181	96.8	71	3.2	0 (0.0)	
**18-24 Months**	2258	2173	96.2	85	3.8		
**24-59 Months**	11538	11473	99.4	65	0.6		
**Total (0–59 Months)**	17501	17274	98.7	226	1.29	1	0.01
**Gender**^b^ **X**^**2**^**: 4.7, df = 1, p = 0.29**							
**Male**	8967	8867	98.9	100	1.1		0.00
**Female**	8534	8407	98.5	126	1.49	1	0.01
**Total**	17501	17274	98.7	226	1.29	1	0.01
**Nationality**^b^ **X**^**2**^**: 72.6, df = 1, p < 0.001**							
**Omani**	17023	16823	98.8	199	1.19	1	0.01
**Non-Omani**	478	451	94.4	27	5.6	0	0
**Total**	17501	17274	98.7	226	1.29	1	0.01
**Governorate**^b^ **X**^**2**^**: 106.1, df = 10, p < 0.001**							
**Buraimi**	399	389	97.5	10	2.5	0	0
**Dakhiliyah**	2568	2555	99.5	13	0.5	0	0
**Dhahira**	958	953	99.5	5	0.5	0	0
**Dhofar**	1384	1335	96.5	49	3.5	0	0
**Musandam**	185	185	100.0	0	0.0	0	0
**Muscat**	3709	3640	98.1	69	1.9	0	0
**North Batinah**	3476	3434	98.8	42	1.2	0	0
**North Sharqiyah**	1253	1248	99.6	4	0.32	1	0.08
**South Batinah**	1869	1852	99.1	17	0.9	0	0
**South Sharqiyah**	1499	1488	99.3	11	0.7	0	0
**Wusta**	201	195	97.0	6	3.0	0	0
**Total**	17501	17274	98.7	226	1.29	1	0.01

^a^ Hepatitis A vaccine at 24 months was not included in the full vaccination analysis because it was introduced in January 2020 which affects the middle age group children coverage in our survey.

^b^* Single case of unvaccinated was included in the partial vaccination category for Chi-square test analysis purpose

The fully vaccinated coverage was significantly higher among different age groups, nationality and governorates when compared to partially vaccinated coverage. The fully vaccinated coverage between males and females was nearly identical, with no significant statistical difference (X^2^: 4.7, df = 1, p = 0.29) as shown in Table 2.

The partially vaccinated were further divided according to number of vaccine doses missed as shown in Table 3. The majority of the children missed only 1 out of the targeted vaccine doses for that age group and nearly 54.9% and 30.5% missed the 18 and 12 months dose respectively.

**Table 3:** Partial vaccination coverage for 4 stages of vaccines according to missed doses.

Partially vaccinated <5 years children (N = 226)								
***Age range*** ^***a***^	**Missed 1 out of the targeted age group vaccine dose**					**Missed 2 out of the targeted age group vaccine dose**		**Missed 3 out of the targeted age group vaccine dose**
	**Total Children**	**Missed birth dose**	**Missed 6 months dose**	**Missed 12 months dose**	**Missed 18 months dose**	**Missed 6 and 12 months dose**	**Missed 12 and 18 months dose**	**Missed 6, 12 and 18 months dose**
	**No. (%)**							
**< 12 months**	5		5					
**12-18 Months**	71		4	66		1		
**18-24 Months**	85	2		1	74		8	
**24-59 Months**	65	2	1	2	50		9	1
***Total*** ***(0–59 Months)***	226 (100%)	4 (1.8%)	10 (4.4%)	69 (30.5%)	124 (54.9%)	1 (0.4%)	17 (7.5%)	1 (0.4%)

^a^Four targeted vaccines age groups:

1. At birth (BCG, HBV)

2. At 6 months (Penta, OPV)

3. At 12 months (MMR1, Varicella)

4. At 18 months (MMR2, DTP & OPV Booster)

The National administrative coverage of 2022 and the current national immunization survey coverage of 2023 were similar for most of the vaccines (at birth 100.0 and 99.9%, at 6 months 99.5 and 99.9%, at 12 months 98.5 and 99.5%, at 18 months 98.5 and 98.9%, at 24 months 96.0 and 94.4%).

The detailed coverage analysis by governorate indicated that crude vaccination coverage for various vaccines was generally high (around 99%) across most governorates, Dhofar (89.3%) and Muscat (92.4%), which showed slightly lower coverage for vaccines targeted at 24-month age group. Common reasons for non-vaccination included medical issues (83.5%), lack of motivation (71.4%), obstacles (38.5%), and insufficient information (47.6%), particularly noted in the Dakhiliyah, Dhahira, North Sharqiyah, and Muscat governorates respectively.

District-wise coverage analysis was nearly uniform ranging from 98–100% for the birth, 6, and 12-month doses across various wilayats/districts. Coverage for the 18-month dose ranged from 97–100%, although Mahout (93.2%) in Al-Wusta governorate and Sadah (95.5%) and Al-Mazyunah (96.6%) districts in Dhofar governorate reported marginally lower coverage compared to other districts. The Hepatitis A vaccine coverage varied significantly, ranging from 82–100%, with the lowest rates observed in Rakhyut (82.4%) and Shaleem Al-Hallaniyat (84.2%) districts.

The factors associated with being fully and partially vaccinated are presented in Table 4 logistic regression analysis. There was only one child in the unvaccinated group hence not included in the analysis. The binary logistic regression revealed that gender was not a significant factor in fully vaccinated coverage [OR 1.3; 95% CI, 0.99–1.7, p > 0.05]. The full immunization coverage was significantly lower among children aged 18–24 months [OR 1.2; 95% CI, 0.4–3.25, p > 0.05] compared to other age groups. Omani children were significantly more likely to be fully vaccinated compared to non-Omani children [OR 4.8; 95% CI, 3.1–7.4, p < 0.001]. Geographically, children in Muscat, Dakhiliyah and North Batinah had lower odds of being fully vaccinated compared to other governorates.

**Table 4:** Factors associated with full and partially vaccinated children- logistic regression analysis.

Associated Factors	Degree of freedom (df)	Significance	Odds Ratio	95% Confidence Interval (CI)	
				Lower	Upper
**Age range (Months)**	3	.00			
**< 12 (Reference)**					
**12-18**	1	.00	.07	.02	.26
**18-24**	1	.71	1.21	.45	3.25
**24-59**	1	.00	5.60	2.43	12.87
**Governorate** ^a^	9	.00			
**Buraimi (Reference)**					
**Dakhiliyah**	1	.73	.83	.30	2.33
**Dhahira**	1	.00	.16	.06	.44
**Dhofar**	1	.00	.17	.05	.56
**Muscat**	1	.69	1.19	.50	2.82
**North Batinah**	1	.26	.61	.26	1.44
**North Sharqiyah**	1	.04	.40	.17	.95
**South Batinah**	1	.00	.13	.04	.43
**South Sharqiyah**	1	.01	.30	.12	.76
**Wusta**	1	.00	.24	.09	.65
**Gender**					
**Male (Female – Reference)**	1	.06	1.3	.99	1.7
**Nationality**					
**Omani (Non-Omani Reference)**	1	.00	4.8	3.1	7.39
**Birth Cohort Year**	4	.00			
**2018 (Reference)**					
**2019**	1	.00	.14	.04	.42
**2020**	1	.00	.06	.02	.20
**2021**	1	.00	.09	.03	.27
**2022**	1	.00	.11	.05	.21

^a^For governorate analysis Musandam was not included because of zero partially vaccinated individuals

## Discussion

In this study, we used the probability proportional to size cluster sampling method, recommended by WHO, to evaluate national vaccination coverage [10]. The study included sub-national populations and less populated, hard-to-reach areas in the sample to enhance representation. This approach led to more comprehensive results, ensuring that the findings accurately reflected vaccination coverage across different districts and the outcomes can be generalized to the entire population.

In this study, mothers were the primary respondents, and most children received their vaccinations at government facilities probably due to their availability at home. This trend, common in many parts of the world, highlights mothers’ pivotal role in ensuring healthcare access for their children, as well as the widespread implementation of government-mandated immunization programs [12].

Oman, similar to China (99%) and Iran (93%) demonstrated high retention (99%) of vaccination cards among its population [13,14]. This practice aligns with the latest WHO recommendations, which emphasize that vaccine coverage studies should be based on written and reliable documentation. In contrast, a study in Congo, Saudi Arabia, Pakistan and Angola had 90, 88, 66.2 and 48% of children had their vaccination cards available at the time of interview respectively [10,15–18].

Gender had no significant effect on vaccination coverage in our study similar to Iran [14]. Our study found that routine national vaccination coverage in children in Oman is nearly 100%, significantly surpassing the global target of 90% [2]. This high coverage aligns with WHO’s administrative reports for Oman over the past decades [19]. Oman’s vaccination rate is comparable to countries like China (99%) Iran (97.8%), USA (>90%), Saudi Arabia (>90%) and India (90.8%) (Indian data is from a provincial study). However, vaccination rates were much lower in Turkey (84.5%), Pakistan (76.5%), Somalia (45.2%) and Angola (37%), highlighting disparities in public health achievements among these nations. Globally, many countries have made significant progress in increasing vaccination coverage since the 1980s. However, despite these improvements, many countries still fall short of the target of 90% coverage [13,14,16–24].

Fully vaccinated coverage was higher in our study compared to a study conducted by Farag MK which was around 86% [16]. This could be due to a strong immunization program, good defaulter retrieval system, accessibility of vaccines, and acceptance by parents. Expatriate children and certain districts of Dhofar governorate had significant partial vaccination coverage probably due to delay in vaccination due to vaccination elsewhere and medical reasons respectively. The Dhofar governorate issue is probably due to Yemeni children in Dhofar, especially in Al-Mazyoonah [25].

The national vaccination coverage in Oman was nearly identical to the reported administrative coverage, indicating the presence of a strong and reliable reporting system. This was further validated by Oman’s top score in the Effective Vaccine Management assessment in 2016 [19,26].

The vaccination coverage was marginally lower during the COVID-19 pandemic birth cohort compared to earlier years in our study, similar to findings from Iraq and as indicated in the global burden study. This decline highlights the disruptions caused by the pandemic, which affected vaccination schedules and coverage globally [24,27].

The national immunization coverage survey faced several challenges. The primary issue was data entry errors during fieldwork, which were corrected during analysis by referring to uploaded immunization cards. Additionally, missed opportunities arose as many potential confounding factors related to vaccine dropouts were not included in the survey due to administrative and logistical limitations. While the survey was generally well-received, some field staff encountered non-cooperation from the public, presenting a challenge in data collection.

Based on this study results, we recommend strengthening routine immunization in low coverage governorates especially in Muscat and Dhofar particularly in the 24-month age group. Develop culturally appropriate educational programs ensuring parents understand the importance of timely and partial immunization. Design specific strategies to improve vaccination uptake among expatriate children, such as mobile vaccination units or outreach programs in communities with a high non-Omani population. Establish clear guidelines for rescheduling vaccinations in cases of mild illness to minimize unnecessary delays in immunization. Implementing these recommendations can help to enhance vaccination coverage and partial vaccination will ultimately improve child health outcomes. The expatriate population with immunization coverage < 95% is considered a high-risk group for possible future outbreaks. As part of the study’s recommendations, it is advised that advocacy, communication, and social mobilization (ACSM) activities related to immunization be actively implemented during World Immunization Week to enhance public awareness and community engagement.

## Conclusions

In conclusion, this study confirms that Oman has achieved high national immunization coverage, with rates exceeding 95%, aligning with WHO targets and administrative reports. Delayed immunization, primarily due to medical reasons, highlights the need for improved scheduling and awareness. Strengthening immunization programs, enhancing follow-up systems, and promoting community education will be crucial to maintaining and improving vaccination coverage. These findings emphasize the effectiveness of Oman’s immunization strategy while identifying areas for targeted interventions to ensure sustained public health success.

## Supporting information

S1 Data NIC Coverage Survey - Data Availability. (XLSX)
